# Colorectal Adenoma Risk Is Increased among Recently Diagnosed Adult Celiac Disease Patients

**DOI:** 10.1155/2018/6150145

**Published:** 2018-04-23

**Authors:** Juan Lasa, Astrid Rausch, Luis Florez Bracho, Josefina Altamirano, Daniela Speisky, María Teresa García de Dávila, Alejandro Iotti, Ignacio Zubiaurre

**Affiliations:** ^1^Gastroenterology Department, British Hospital, Buenos Aires, Argentina; ^2^Pathology Department, British Hospital, Buenos Aires, Argentina

## Abstract

**Background:**

The association between celiac disease and colorectal neoplasia has been previously studied, but the question whether recently diagnosed celiac patients show an increased colorectal adenoma prevalence remains unanswered.

**Aims:**

To compare the prevalence of colorectal adenomas between adult patients with a recent diagnosis of celiac disease versus healthy controls.

**Materials and Methods:**

A retrospective case-control study was undertaken. Patients with a diagnosis of celiac disease at an age of 45 years or more who undertook colonoscopy six months before or six months after the initiation of a gluten-free diet were enrolled as cases. Asymptomatic subjects undertaking screening colonoscopy were recruited as controls in a 2 : 1 fashion. The prevalence of colorectal adenomas and the prevalence of advanced adenomas were compared between groups.

**Results:**

57 celiac disease patients and 118 controls were enrolled. There was a greater prevalence of female patients among the celiac group, with no significant differences in terms of age. There were more obese patients among controls and a higher proportion of tabaquism among celiac patients. Adenoma prevalence was significantly higher among celiac patients (47.37% versus 27.97%, *p* = 0.01). Advanced adenoma detection was not different between groups.

**Conclusion:**

Adult patients with a recent diagnosis of celiac disease have an increased prevalence of colorectal adenomas.

## 1. Background

Celiac disease is a relatively common autoimmune disorder triggered by the intestinal exposure to gluten—a glycoprotein present in wheat, barley, rye, and oat [1]. Classically, it has been described as a condition causing malabsorption of nutrients, with diarrhea or failure to thrive as common clinical features among pediatric patients. Atypical forms of presentation however may be more common in adult patients with celiac disease. As a consequence, clinical elements such as iron-deficiency anemia or osteoporosis can be the initial features behind the aforementioned disorder [2].

One of the most relevant issues regarding celiac disease is the risk of developing both malignant and nonmalignant tumors [3]. It has been well described its association with an increased risk of small-bowel adenocarcinoma as well as lymphoproliferative disorders—such as enteropathy-associated T-cell lymphomas [4]. Celiac disease has been linked to extraintestinal malignant tumors, such as esophageal squamous-cell carcinoma. Interestingly, there is a relative scarcity of evidence assessing the risk celiac disease patients exhibit in developing colorectal neoplasia. According to a meta-analysis by Han et al. [5], there was no significant association between these two entities. It should be noted however that most of the studies that assess a possible link between celiac disease and colorectal cancer are retrospective, do not always have a valid comparator, and show a high variability in terms of the way celiac disease is defined: by means of serological findings only or biopsy-based diagnosis.

Most colorectal cancers derive from benign asymptomatic neoplastic lesions known as adenomas [6]. These lesions can be detected and effectively treated before their progression to adenocarcinoma, and recent evidence shows that polypectomy effectively decreases the risk of developing colorectal adenocarcinoma. There is a myriad of risk factors that are behind the development of colorectal adenomas. Identifying such factors is important, since they become crucial in the decision-making process of preventive measures such as screening colonoscopy [7]. The question whether celiac disease may per se increase the risk of colorectal adenomas has been assessed before, showing no significant association. However, most of the adult patients included in these studies were already diagnosed and following a gluten-free diet [8–10]. The question whether recently diagnosed—and, as a consequence, untreated—celiac disease could imply a significant risk of colorectal adenomas has not been answered. Hence, we sought to evaluate the prevalence of colorectal adenomas among recently diagnosed celiac disease patients compared to nonceliac, otherwise healthy, controls.

## 2. Materials and Methods

### 2.1. Study Design and Population

We conducted a retrospective case-control study at our Gastroenterology Department. Medical records from January 2010 to July 2017 as well as from both Endoscopy and Pathology Departments were reviewed. Patients with a diagnosis of celiac disease at an age of 45 years or older were initially screened. Those patients with a colonoscopy performed between 6 months before or after the diagnosis of celiac disease and the initiation of a gluten-free diet were considered for inclusion as cases.

Celiac disease was defined as the presence of serum IgA or IgG antitissue transglutaminase antibodies and a small-bowel biopsy showing some degree of villous atrophy along with an abnormal increase of intraepithelial lymphocytes (more than 25 intraepithelial lymphocytes per 100 epithelial cells) (Marsh 3A to 3C). Asymptomatic subjects undertaking screening colonoscopy were randomly recruited as controls in a 2 : 1 fashion. Randomization was computer-generated using our Endoscopy Unit's database.

The study protocol was approved by our Institution's Internal Review Board (date of approval: April 5, 2017, protocol number #735HB). Since it was a retrospective study, no informed consent was necessary for each patient that was enrolled. The study protocol conforms to the ethical guidelines of the 1975 Declaration of Helsinki.

### 2.2. Outcome Measures

The prevalence of colorectal adenomas and/or colorectal cancer as well as their location throughout the colon were compared between groups. Additionally, the prevalence of advanced adenomas was compared. We defined advanced adenoma as any adenomatous lesion with at least one of the following features: (a) a predominant villous component, (b) lesion diameter over 10 mm, and (c) the presence of high-grade dysplasia. Location of the adenomatous lesions found was classified as right-sided adenomas if they were proximal to the splenic flexure. Accordingly, adenomas located distal to the splenic flexure were regarded as left-sided lesions.

Colorectal neoplasia risk factors were also compared between groups: age, gender, familiar history of colorectal neoplasia, tabaquism, obesity, and diabetes. Tabaquism was defined as the consumption of at least 5 cigarettes or its equivalent for at least 6 months: current smokers as well as subjects with a history of smoking were contemplated under this definition. Obesity was defined as any subject with a body mass index > 30. Diabetes was defined as a serum glucose level of at least 126 mg/dl or an abnormal glucose tolerance test or being under treatment with insulin and/or other medication used for diabetes mellitus treatment. Colonoscopy-related features were also recorded and subsequently compared between groups: cecal intubation, colonoscope withdrawal time, and bowel cleansing quality assessed by the Boston Bowel Preparation Scale [11]. Colonoscopies were performed by experienced endoscopists with an adenoma detection rate higher than 20%, using high-definition endoscopes. These variables are systematically registered in the Endoscopy Department database.

### 2.3. Statistical Analysis

Assuming an alpha error of less than 5% and a power > 80%, we hypothesized that celiac disease patients would have a colorectal adenoma prevalence of 40% versus 20% in the case of control subjects; considering that two controls would be enrolled for each case, we estimated that 58 cases along with 116 controls would be needed.

Stata software was used for the statistical analysis (v11.1, StataCorp, College Station, Texas, USA). Categorical variables were described as percentages with their corresponding 95% confidence interval (95% CI). Numerical variables were described as median with their range. For the comparison of categorical variables, Fisher test was used; for the comparison of numerical variables, Mann–Whitney test was used.

A univariate analysis was performed with odds ratio (OR) and their 95% CI calculation to evaluate variables significantly associated with the presence of colorectal adenomas as well as advanced adenomas. Finally, a multivariate analysis including all variables with a *p* value of less than 0.1 on univariate analysis was performed to identify independent variables associated with both adenoma and advanced adenoma prevalence.

## 3. Results

During the study period, 323 adult patients with a diagnosis of celiac disease were identified; however, only 57 celiac patients fulfilled inclusion criteria. Accordingly, 118 healthy subjects undergoing screening colonoscopy were randomly selected and regarded as controls.

All of the celiac disease subjects had their colonoscopy either 6 months prior to the diagnosis of celiac disease (35%) at the moment of diagnosis (14%) or 6 months after the diagnosis (51%). The main clinical findings of the celiac disease patients at diagnosis were chronic diarrhea (28%), iron-deficiency anemia (22.8%), abdominal bloating (17.5%), and a combination of chronic diarrhea and anemia (8.7%); 12% were asymptomatic at diagnosis. Colonoscopy among celiac disease patients was performed during the work-up of chronic diarrhea and/or iron-deficiency anemia or for screening purposes. There was no difference in terms of adenoma risk regarding symptom profile at celiac disease diagnosis.

Table 1 shows the comparison of the main clinical and endoscopic features between cases and controls. We found a higher proportion of female subjects in the celiac disease group, as well as a lower prevalence of obesity among these patients. These findings were not surprising, considering the fact that celiac disease is more common among women and obesity may not be as frequent as in a nonceliac population. On the other hand, tabaquism was found to be more frequent among celiac disease patients (29.8% versus 15.2%, respectively, *p* = 0.02).

It is worth mentioning that we found no significant differences in terms of colonoscopy quality indicators between groups, such as cecal intubation rate, endoscopic withdrawal time, and bowel cleansing quality.

Table 2 shows the comparison of the endoscopic findings between groups. Overall adenoma detection was 34.2%. Adenoma detection was significantly more frequent among celiac disease patients (47% versus 28%, *p* = 0.01). However, advanced adenoma detection was not different between groups (10.5% versus 8.4%, *p* = 0.65). Additionally, we found a significant difference in terms of adenoma distribution: among celiac disease patients, a higher proportion of left-sided adenomas was found (36.8% versus 17.8%, *p* = 0.006). We did not find any colorectal cancer among the subjects included for study. Additionally, no other neoplastic disease was identified.

On multivariate analysis (Table 3), celiac disease remained a variable significantly associated with an increased odd of adenoma prevalence, as well as left-sided adenoma prevalence. Celiac disease failed to show significant odds of advanced adenoma on multivariate analysis.

## 4. Discussion

According to our results, recently diagnosed celiac disease among adult patients is significantly associated with increased odds of colorectal adenomas, in particular left-sided adenomas. These results are relevant since they may highlight an increased risk of colorectal adenoma development among adult celiac disease patients.

As stated before, there are some relevant points to be highlighted regarding the evidence on the link between celiac disease and colorectal neoplasia. First of all, there is a considerable amount of publications that fail to show an association between celiac disease and an increased risk of colorectal cancer [12–15]. This is not the case of other malignant tumors that are located outside the small bowel, such as esophageal squamous-cell carcinoma. As a consequence, the possibility that—due to a mechanism that may be related to malabsorption, abnormal inflammatory response, or even the effect of gluten on itself in this particular population—celiac disease may provide an increased risk of neoplasia outside the small bowel is biologically plausible.

When it comes to the evidence trying to establish the possible link between celiac disease and colorectal adenomas, there is a relatively scarce amount of publications [8–10]. However, these studies failed to show an increased risk of colorectal adenomas among celiac patients. It is important to highlight some common features of these publications: all of them included already diagnosed celiac disease patients following a gluten-free diet and compared them to nonceliac, otherwise healthy, controls. Noteworthy, Pereyra et al. [10] suggested, through a subgroup analysis, that among celiac disease patients without a good compliance of gluten-free diet, the risk of colorectal adenomas was increased. Even though the proportion of such patients without a good adherence to a gluten-free diet was low, this finding in fact raises the possibility that untreated celiac disease patients may show an increased risk of colorectal neoplasia.

The question behind this finding by Pereyra et al. [10] was whether the lack of a gluten-free diet among celiac patients could be a trigger to the development of colorectal lesions. This possibility can be furtherly supported by the findings of increased risk of mortality among recently diagnosed celiac disease patients [16]. Among the physiological mechanisms that could underlie such association, it should be noted that among adult patients with a recent diagnosis of celiac disease, micronutrient deficiencies are a common feature. As a consequence, it becomes plausible that a nutritional factor could contribute to some extent to the genesis of neoplasms among celiac disease patients—including colorectal neoplasia. Additionally, it may highlight the relevance of gluten-free diet compliance as a potentially relevant protective factor against neoplasia development.

Our study design was oriented towards this assumption. We chose a population that has a logical exposure to gluten: those patients with symptomatic, recently diagnosed celiac disease. Hence, the evaluation of the colon had to be framed near the time of diagnosis. Colonoscopy among these patients showed increased odds of colorectal adenomas, especially those adenomas located in the left colon. Furthermore, although it did not reach a statistical significance, we observed a tendency towards increased odds of advanced adenomas among celiac disease subjects. When comparing these findings to the abovementioned evidence on the matter, it could be argued that the increased risk that we found could be due to gluten exposure that the celiac patients in our study naturally had or a nutritional deficiency-related factor as a consequence of intestinal malabsorption as aforementioned. As a consequence, it could be hypothesized that persistent gluten exposure among already diagnosed celiac disease patients could raise the risk of colorectal neoplasia.

Another strength showed by our study is the emphasis made on colonoscopy quality indicators. It is noteworthy that, on previously published experiences on the subject, the overall adenoma detection rates were relatively low: in any case, the prevalence of adenomas was over 20%. Since all of these studies are retrospective, there may be a relative lack of information regarding the quality features that are important features to be analyzed when colorectal adenoma prevalence is assessed. Our Endoscopy Unit has collected in its database as many features regarding quality indicators as possible, allowing us to show a rather complete profile of the colonoscopies that were included for analysis. This is reflected in the adenoma prevalence among nonceliac controls: 27.97%, which is significantly higher than the prevalence showed in previously published studies.

We failed to show a relationship between the pattern of symptoms at celiac disease diagnosis and colorectal adenoma prevalence. We also failed to show a significant association between colorectal adenomas and other well-established risk factors, such as tabaquism and family history of colorectal neoplasia. These results may be due to the lack of adequate power of our study to show such results. We did find an association between age and colorectal adenoma risk.

Some limitations must be mentioned. Firstly, this is a retrospective study, with all the limitations that are implicated in such studies. Additionally, a relevant clinical feature could not be measured, due to the retrospective nature of the study: the time from initiation of symptoms to diagnosis among celiac disease patients. It has been suggested that, among adult celiac patients, a delay in the diagnosis could be related to worse outcomes. This could have been an important information that would have provided more relevance to our study. We did not choose gender-matched control subjects—only age-matched—a factor that could have a significant influence on the results on prevalence of colorectal neoplasia; however, gender differences failed to show a significant influence on colorectal neoplasia according to multivariate analysis. It is also worth mentioning that this study was not a multicenter study. Last but not least, it should be noted that celiac disease patients who underwent colonoscopy did so in many cases due to symptoms such as diarrhea or anemia—a feature that could partially explain the high proportion of colorectal adenomas diagnosed. However, this study shows the most common clinical scenarios in which celiac disease is diagnosed among adults and as a consequence may be representative of a real-life setting.

In conclusion, colorectal adenoma prevalence was shown to be increased among recently diagnosed adult celiac disease patients, with a special increase in the prevalence of left-sided lesions. More evidence is needed to determine whether long-term exposure to gluten among celiac patients may result in an increased risk of colorectal adenoma and, thus, subsequent adenocarcinoma development.

## Figures and Tables

**Table 1:** Comparison of the main characteristics of celiac disease patients and controls.

	Celiac disease patients (%, *n*/*N*)	Control subjects (%, *n*/*N*)	OR (95% CI)	*p*
Gender (male)	26.3 (15/57)	59.3 (70/118)	0.24 (0.11–0.51)	<0.001
Age	58 (49–75)	57 (50–64)	N/A	0.9
Familiar history of colorectal neoplasia	24.5 (14/57)	28 (33/118)	0.83 (0.41–1.73)	0.6
Obesity	1.7 (1/57)	21.2 (25/118)	0.06 (0.008–0.54)	0.001
Diabetes	3.5 (2/57)	7.6 (9/118)	0.44 (0.09–2.12)	0.3
Tabaquism	29.8 (17/57)	15.2 (18/118)	2.33 (1.08–5.03)	0.02
Colonoscopy characteristics				
Cecal intubation	96.5 (55/57)	97.5 (115/118)	0.71 (0.11–4.44)	0.7
B-BPS	7 (5–9)	7 (6–9)	N/A	0.2
Withdrawal time	6 (5–10)	7 (6–9)	N/A	0.3

B-BPS: Boston Bowel Preparation Scale.

**Table 2:** Comparison of endoscopic findings between groups.

	Celiac disease patients (%, *n*/*N*)	Control subjects (%, *n*/*N*)	OR (95% CI)	*p*
Adenoma	47.4 (27/57)	28 (33/118)	2.31 (1.18–4.53)	0.01
Left-sided adenoma	36.8 (21/57)	17.8 (21/118)	2.69 (1.29–5.61)	0.006
Right-sided adenoma	17.5 (10/57)	15.2 (18/118)	1.18 (0.5–2.76)	0.7
Advanced adenoma	10.5 (6/57)	8.5 (10/118)	1.27 (0.43–3.71)	0.6

**Table 3:** Multivariate analysis of variables associated with the presence of colorectal adenomas.

	OR (95% CI)	*p*
Celiac disease	2.95 (1.36–6.41)	0.006
Gender	1.38 (0.68–2.81)	0.36
Age	1.02 (1–1.04)	0.016
Obesity	1 (0.36–2.71)	0.98
Tabaquism	0.89 (0.38–2.03)	0.77

## Additional Points

*Key Summary*. (i) Potential association between increased colorectal adenoma prevalence and treated celiac disease patients has been previously assessed, showing no significant association. (ii) In our study, we assessed whether recently diagnosed, untreated adult celiac patients may have increased odds of colorectal adenomas on colonoscopy. (iii) A significant increase in colorectal adenoma prevalence was found among recently diagnosed celiac disease patients not following a gluten-free diet. (iv) This finding suggests that adult celiac patients should undergo screening colonoscopy at diagnosis.
